# Neuroprotective Potential of Betulin and Its Drug Formulation with Cyclodextrin—In Vitro Assessment

**DOI:** 10.3390/ijms26125605

**Published:** 2025-06-11

**Authors:** Agnieszka Zakrzeska, Agnieszka Kołodziejczyk, Piotr Komorowski, Paulina Sokołowska, Bożena Rokita, Radosław A. Wach, Małgorzata Siatkowska

**Affiliations:** 1 Department of Nursing, University of Medical Science in Bialystok, Krakowska 9, 15-875 Bialystok, Poland; a.zakrzeska@wsmed.pl; 2 Laboratory of Molecular and Nanostructural Biophysics, Bionanopark, 114/116 Dubois, 93-465 Lodz, Poland; p.komorowski@bionanopark.pl; 3 BioMatGel, Pienista 41F/11, 94-109 Lodz, Poland; bozena.rokita@biomatgel.pl (B.R.); radoslaw.wach@biomatgel.pl (R.A.W.); 4 Department of Pharmacology and Toxicology, Medical University of Lodz, 7/9 Zeligowskiego, 90-752 Lodz, Poland; paulina.sokolowska@umed.lodz.pl

**Keywords:** differentiated neuroblastoma cells, betulin, SH-SY5Y, neuroprotection, ROS, apoptosis

## Abstract

Central nervous system disorders, such as Alzheimer’s disease, Parkinson’s disease, and multiple sclerosis, are associated with complex pathophysiological mechanisms involving oxidative stress, inflammation, and protein misfolding. According to the literature, betulin, a natural compound derived from the bark of birch trees, demonstrates promising neuroprotective effects. This study investigates the neuroprotective potential of betulin and its complex with cyclodextrin, referred to as Betula Forte, using an in vitro model of differentiated SH-SY5Y neuroblastoma cells. Specifically, the study explores the antioxidant and antiapoptotic properties of these compounds under oxidative stress induced by hydrogen peroxide (H_2_O_2_). Our results indicated that Betula Forte exhibited lower cytotoxicity compared to betulin alone. Both substances enhanced cell viability in pre-incubation and co-incubation models, with Betula Forte showing superior efficacy under severe oxidative stress. Additionally, both substances exhibited protective effects against H_2_O_2_-induced oxidative stress and apoptosis, as evidenced by reduced levels of reactive oxygen species (ROS) and a lower number of apoptotic cells compared with the H_2_O_2_-treated cells. These findings suggest that Betula Forte, due to lower cytotoxicity, may offer a more effective neuroprotective strategy than betulin alone, highlighting its potential as a therapeutic agent for neurodegenerative diseases.

## 1. Introduction

Disorders of the central nervous system may manifest as movement disorders (Parkinson’s disease, Huntington’s disease), dementia syndromes (Alzheimer’s disease), demyelinating diseases (multiple sclerosis), vascular diseases (stroke), and other conditions [1]. These disorders exhibit diverse and complex mechanisms that are interconnected and often exacerbate one another, creating a vicious cycle that drives disease progression. Key mechanisms may involve misfolded proteins, such as beta-amyloid in Alzheimer’s disease or alpha-synuclein in Parkinson’s disease, as well as oxidative stress, neuroinflammation, mitochondrial dysfunction, dysregulated calcium homeostasis, genetic mutations, and other contributing factors [1,2]. The search for new substances capable of targeting pathophysiological mechanisms to halt or significantly slow disease progression remains a current challenge for medicine and science.

Natural products have long been considered a valuable source of pharmacologically active compounds, particularly for treating cancer and infectious diseases. Despite the wide availability of synthetic pharmaceuticals, natural products still play a significant role in the prevention and treatment of diseases due to their natural diversity, complexity, uniqueness, low toxicity, and wide-ranging effectiveness [3]. An increasing body of evidence suggests that active compounds of plant origin such as derivatives of pentacyclic triterpenes may represent potential therapeutic candidates for brain disorders [4,5,6]. Triterpene compounds are typically found in the bark, cork, resin, and waxy coating of leaves and flowers, serving a protective function against insect and microorganism attacks. One of the natural sources of lupane triterpenes is the outer layer of the bark of the white birch species (Betula), such as B. verrucosa, B. pendula, B. pubescens, and B. alba. Various sources report that some of the main components of birch bark are betulin (up to 35%) and lupeol (5–10%). Betulin forms a stable suspension with oils and fats, mixes easily with free-flowing ingredients, and remains stable under heat [7]. Betulin, known for its pleiotropic activity, demonstrates anti-inflammatory, antiviral, antibacterial, immunomodulatory, and antitumor properties [3,8]. As such, it holds potential as a promising therapeutic agent for the treatment of various diseases.

To enhance the solubility and bioavailability of betulin, we developed a new formulation named Betula Forte as a complex of betulin with cyclodextrin in a 1:2 molar ratio (betulin:cyclodextrin). This stoichiometric ratio was considered when calculating betulin-equivalent doses in the current study, ensuring a valid and accurate comparison between Betula Forte and pure betulin.

In our previous study, we demonstrated the neuroprotective potential of betulin and betulin in complex with cyclodextrin in a rat model of Alzheimer’s disease (AD) [9]. Betulin, particularly in the form of its complex with cyclodextrin, improved spatial memory in rats with AlCl3-induced AD. The proposed mechanism of action included a reduction in the level of AD-related proteins, i.e., Aβ1-42 and APLP2, while also decreasing the expression of pro-inflammatory factor TNF-α in the rat brain. This study suggests that betulin and its improved formulation could serve as a basis for developing new, effective prophylactic drugs for AD and possibly other neurological disorders; however, to confirm this hypothesis, further research is needed.

Therefore, the primary aim of the present study was to evaluate the neuroprotective properties of a novel betulin–cyclodextrin complex, under the name Betula Forte, in comparison to pure betulin. To achieve this, we utilized an established in vitro model of differentiated neuroblastoma SH-SY5Y cells, which serve as a reliable in vitro model for studying neuronal function, differentiation, and pathology. Given that oxidative stress and apoptosis are key, interrelated mechanisms contributing to the progression of various central nervous system (CNS) disorders, this study further aimed to assess whether Betula Forte offers enhanced antioxidant and antiapoptotic activity relative to betulin alone. Through this comparative approach, we sought to evaluate whether complexation with cyclodextrin could improve betulin’s bioactivity and potential therapeutic relevance in the context of neurodegenerative disease models.

## 2. Results

### 2.1. The Effect of Betulin, Betula Forte, and H_2_O_2_ on Differentiated SH-SY5Y Neuroblastoma Cells

**Figure 1:**
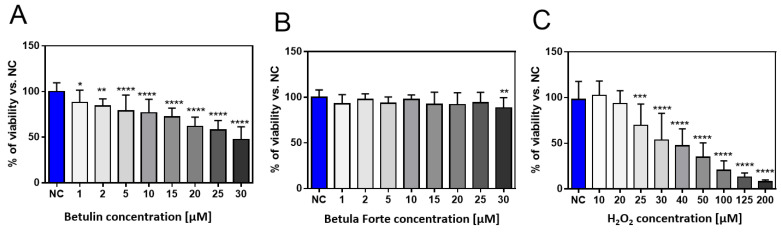
Cell viability after 24 h of incubation of differentiated SH-SY5Y neuroblastoma cells with (**A**) betulin, (**B**) Betula Forte, and (**C**) H_2_O_2_ at various concentrations. Data are presented as means ± standard deviations (SD). The number of independent experiments was as follows: (**A**) *n* = 4, (**B**) *n* = 4, and (**C**) *n* = 5 (six independent measurements were analyzed in each experimental repetition). * indicates significant differences between the negative control (NC) and the tested compounds; * *p* < 0.05, ** *p* < 0.01, *** *p* < 0.005, **** *p* < 0.001.

The effect of betulin and Betula Forte on the viability of differentiated neuroblastoma cells was studied in a wide range of concentrations (from 1 to 30 µM). Additionally, to determine the IC50 and IC80 values for H_2_O_2_, the cytotoxicity of the H_2_O_2_ solution in a specified range toward differentiated neuroblastoma cells was measured. According to the obtained results, betulin caused a decrease in the viability of differentiated SH-SY5Y neuroblastoma cells in a dose-dependent manner and induced a cytotoxic effect above the concentration of 20 µM (Figure 1A).

No cytotoxic effect was observed for Betula Forte in the entire range of tested concentrations (Figure 1B). In turn, the expected dose-dependent effect of H_2_O_2_ on differentiated SH-SY5Y neuroblastoma cells was demonstrated (Figure 1C). Based on the obtained results concerning the viability of neuroblastoma cells exposed to H_2_O_2_, the IC50 and IC80 concentration values were calculated as 40 and 100 µM, respectively.

### 2.2. Assessment of the Effects of Betulin and Betula Forte in the Pre-Incubation Variant

**Figure 2:**
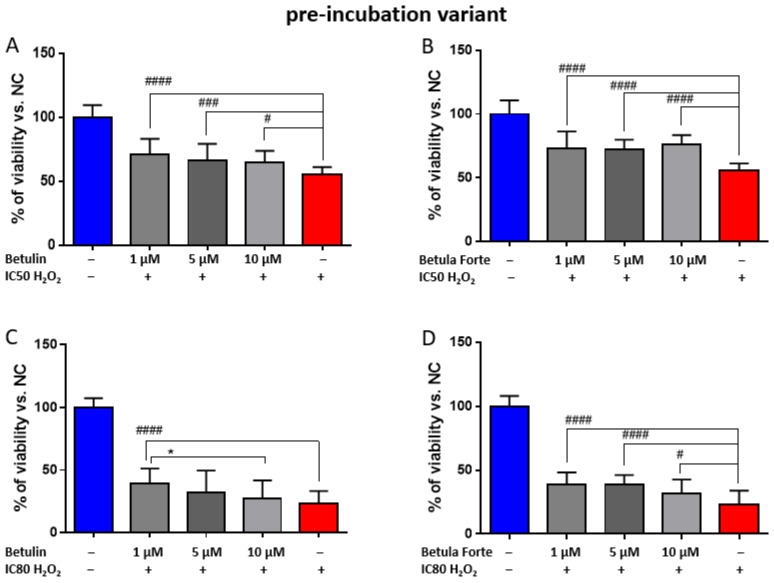
Cell viability after 24 h of incubation of differentiated SH-SY5Y neuroblastoma cells with (**A**,**C**) betulin and (**B**,**D**) Betula Forte in the pre-incubation variant. Data are presented as means ± standard deviations (SD). The number of independent experiments was as follows: (**A**–**C**) *n* = 4, (**D**) *n* = 5 (six independent measurements were analyzed in each experimental repetition). # indicates significant differences between the positive control (PC), i.e., H_2_O_2_ at IC 50 or IC80, and the tested concentrations of betulin or Betula Forte (# *p* < 0.05, ### *p* < 0.005, #### *p* < 0.001). * indicates significant differences between two different concentrations of the tested compound (* *p* < 0.05).

The aim of the pre-incubation variant study was to verify whether active substances such as betulin and Betula Forte exert a protective effect on differentiated neuroblastoma cells against the action of a neurotoxic factor (i.e., H_2_O_2_). In the case of betulin, for the IC50 H_2_O_2_ concentration, a statistically significant protective effect was observed on differentiated SH-SY5Y cells for all selected concentrations (Figure 2A).

The increase in cell viability relative to the cells exposed to H_2_O_2_ at the IC50 concentration ranged from 9.2 to 15.8% (Table 1). In turn, with a strong H_2_O_2_ effect, i.e., at the IC80 level, betulin improved cell viability in the pre-incubation variant in a statistically significant manner only at a concentration of 1 μM (increase in cell viability by 17.5% compared to H_2_O_2_ at IC80; Table 1). For betulin concentrations of 5 and 10 µM and the H_2_O_2_ concentration resulting in IC80 in the pre-incubation variant, no statistically significant changes in cell viability were obtained relative to the PC, although an increased tendency in cell viability is observed.

A stronger neuroprotective effect in the pre-incubation variant was obtained with Betula Forte. At all tested concentrations, i.e., 1, 5 and 10 µM, a statistically significant increase in cell viability was obtained compared to the PC (Figure 2B,D), ranging from 17.5% to 20.5% for IC50 and 8.25% to 15.5% for IC80 (Table 1).

### 2.3. Evaluation of Betulin and Betula Forte in the Co-Incubation Variant

**Figure 3:**
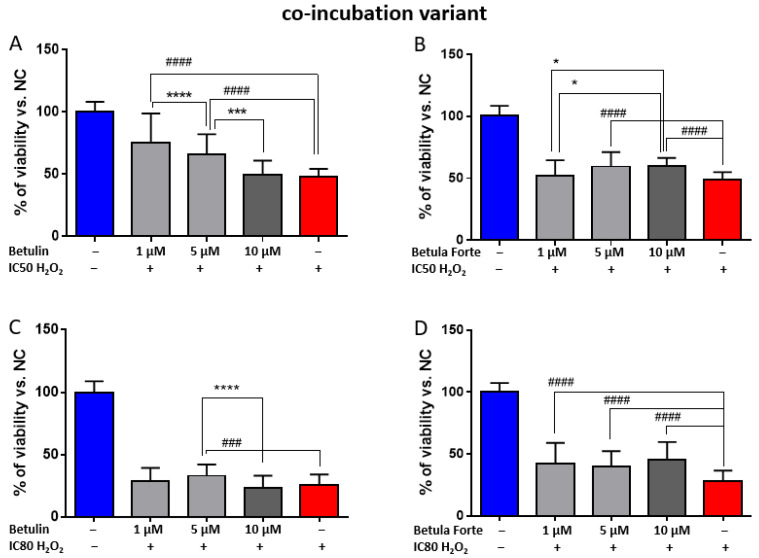
Cell viability after 24 h of incubation of differentiated SH-SY5Y neuroblastoma cells with (**A**,**C**) betulin and (**B**,**D**) Betula Forte in the co-incubation variant. Data are presented as means ± standard deviations (SD). The number of independent experiments was as follows: (**A**) *n* = 4, (**B**) *n* = 4, (**C**) *n* = 7, (**D**) *n* = 5 (six independent measurements were analyzed in each experimental repetition). # indicates significant differences between the positive control (PC), i.e., H_2_O_2_ at IC 50 or IC80, and the tested concentrations of betulin or Betula Forte (### *p* < 0.005, #### *p* < 0.001). * indicates significant differences between two different concentrations of the tested compound (* *p* < 0.05, *** *p* < 0.005, **** *p* < 0.001).

In the co-incubation variant, it was verified whether the active substances exert a protective effect on differentiated SH-SY5Y cells with simultaneous exposure to H_2_O_2_ for 24 h. Betulin in the co-incubation variant caused a statistically significant increase in cell viability for the concentrations of 1 and 5 μM compared to cells incubated with H_2_O_2_ at the IC50 concentration (Figure 3A) by 27.6% and 18.2%, respectively (Table 2). In this case, Betula Forte showed a smaller but statistically significant effect, and the relative increase in cell viability was from 10.7% and 10.9% for the concentrations of 5 and 10 μM, respectively (Table 2).

At a concentration of H_2_O_2_ reducing cell viability by 80% (IC80), no protective effect was obtained for betulin at the concentrations of 1 and 10 μM (Figure 3C). A slight increase in the viability of differentiated neuroblastoma cells (by 7%, Table 2) was observed only at a concentration of 5 μM relative to the PC. In turn, Betula Forte showed more effective action and improved cell viability in relation to the PC in a statistically significant way for all three concentrations, i.e., 1, 5, and 10 μM (Figure 3D), by 13.6%, 11.9%, and 17.4%, respectively (Table 2). The observed effect is stronger than that observed for Betula Forte at the toxin concentration IC50.

### 2.4. Reactive Oxygen Species (ROS) Production and Apoptosis Detection in Differentiated SH-SY5Y Cells in the Co-Incubation Variant

**Figure 4:**
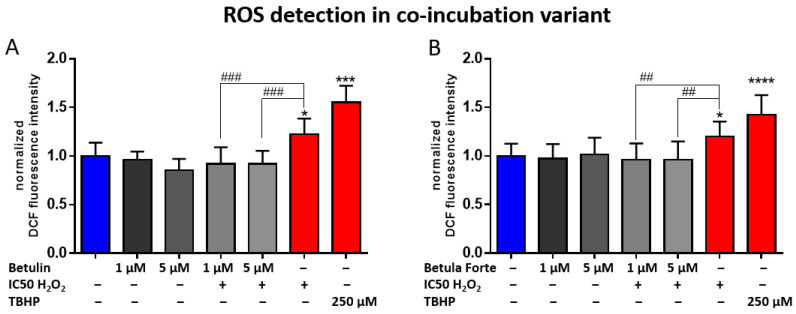
ROS detection in differentiated SH-SY5Y neuroblastoma cells after exposure to (**A**) betulin and (**B**) Betula Forte at various concentrations and during co-incubation with H_2_O_2_ at IC50. Data are presented as means ± standard deviations (SD). The number of independent experiments was as follows: (**A**,**B**) *n* = 3 (six independent measurements were analyzed in each experimental repetition). # indicates significant differences between the ROS generating agent (H_2_O_2_ at IC 50) and the tested compound added simultaneously to the culture (## *p* < 0.01, ### *p* < 0.005). * indicates significant differences between the negative control (NC) and the tested compounds (* *p* < 0.05, *** *p* < 0.005, **** *p* < 0.001).

Based on the analyses of ROS detection, it was demonstrated that H_2_O_2_ at a concentration corresponding to IC50 and tert-butyl hydroperoxide (TBHP) at 250 µM significantly increased the proportion of cells exhibiting ROS activation compared to control cells after 2 h of incubation (Figure 4). In the case of betulin, co-incubation with H_2_O_2_ resulted in a statistically significant reduction in ROS activation for both tested concentrations (1 µM and 5 µM) compared to H_2_O_2_ alone at IC50 (Figure 4A). Similarly, Betula Forte also caused a statistically significant decrease in ROS activation in differentiated SH-SY5Y cells during 2 h of co-incubation at both concentrations tested (1 µM and 5 µM; Figure 4B).

**Figure 5:**
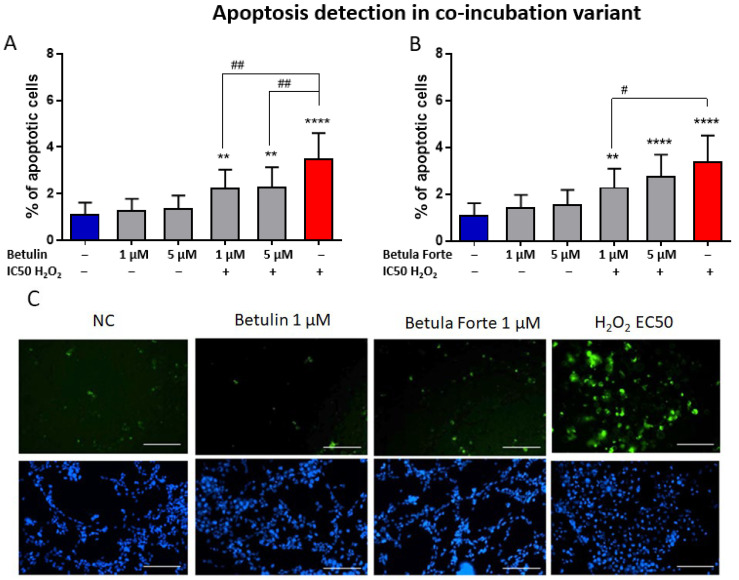
Apoptosis detection in differentiated SH-SY5Y neuroblastoma cells after exposure to (**A**) betulin and (**B**) Betula Forte at various concentrations and during co-incubation with H_2_O_2_ at IC50. (**C**) Fluorescence images of cells stained with Annexin V and Hoechst (scale bar is 100 µm). Data are presented as means ± standard deviations (SD). The number of independent experiments was as follows: (**A**,**B**) *n* = 4 (six independent measurements were analyzed in each experimental repetition). # indicates significant differences between the apoptosis generating agent (H_2_O_2_ at IC 50) and the tested compound added simultaneously to the culture (# *p* < 0.05, ## *p* < 0.01). * indicates significant differences between the negative control (NC) and the tested compounds (** *p* < 0.01, **** *p* < 0.001).

In case of early apoptosis evaluation, neither betulin nor Betula Forte influenced the induction of early apoptosis when administered alone to cultures of differentiated SH-SY5Y neuroblastoma cells compared to negative control cells (Figure 5A–C). Apoptosis naturally occurs at a low level in any cell culture. This baseline level of apoptosis is experimentally defined and depends on the optimization of culture conditions, including the choice of growth medium, supplements, temperature, CO_2_ levels, and humidity. In the present study, the baseline level of early apoptosis for the negative control did not exceed 2%, indicating proper culture optimization. Treatment with H_2_O_2_ at a concentration corresponding to IC50 resulted in more than a twofold increase in the induction of early apoptosis compared to the negative control (Figure 5A,B).

In differentiated SH-SY5Y neuroblastoma cells co-incubated with betulin or Betula Forte and exposed to H_2_O_2_, a reduction in the number of apoptotic cells was observed compared with the positive control (at IC50). Specifically, betulin at concentrations of 1 µM and 5 µM decreased the apoptotic cell numbers by 1.5-fold (Figure 4A) in a statistically significant manner (*p* < 0.01). Similarly, Betula Forte at the same concentrations reduced apoptosis levels by 1.5- and 1.2-fold, respectively (Figure 5B); however the reduction was statistically significant only at 1 μM (*p* < 0.05) when compared to the PC.

## 3. Discussion

Neuroprotection is a multifaceted process involving a wide array of molecular mechanisms aimed at preserving neuronal structure and function in the face of injury or disease. The brain, as a highly dynamic and metabolically demanding organ, is particularly vulnerable to various insults, including oxidative stress, excitotoxicity, inflammation, and mitochondrial dysfunction. Neuroprotective strategies seek to mitigate these challenges by targeting cellular pathways that promote survival, repair, and resilience within the nervous system [1].

In the search for new and effective neuroprotective agents, natural compounds have attracted significant attention due to their multitarget properties and relative safety profiles [10,11,12]. Among these, betulin and betulinic acid—two triterpenoids derived from birch bark—have been widely studied. While betulinic acid has been the subject of extensive research, particularly in oncology and neuroprotection [12,13,14,15], much less is known about the pharmacological properties of betulin itself. Importantly, betulin demonstrates certain advantages over its oxidized derivative, betulinic acid. Its lower polarity allows for better interaction with cellular membranes, potentially improving bioavailability and therapeutic efficacy [16]. Improved formulations of betulin can bring superior advantages in their potential neuroprotective applications.

In this study, we conducted a comprehensive analysis of the ability of betulin and its complex with cyclodextrin referred to as Betula Forte to promote the survival of neuron-like SH-SY5Y cells under various experimental conditions. Referring to the results of our previous work [9], we considered oxidative stress as one of the most impactful consequences of aging, particularly in the context of neurodegenerative disorders. Oxidative stress arises from an imbalance between the production of reactive oxygen species (ROS) and the body’s ability to neutralize these reactive molecules through antioxidants. While ROS are normal byproducts of cellular metabolism and play roles in cell signaling and immune responses, their excessive accumulation can damage proteins, lipids, and DNA. This damage exacerbates cellular dysfunction and accelerates aging processes, particularly in the brain, which is highly susceptible due to its high oxygen consumption and limited regenerative capacity [16,17].

To induce oxidative stress in SH-SY5Y cells, we utilized H_2_O_2_ at two concentrations. The first, i.e., 40 μM, reduced cell viability to 50% (IC50 concentration), while the second, i.e., 100 μM, induced severe stress, reducing cell viability to 20% (IC80 concentration) (Figure 1C). To exclude potentially cytotoxic effects, Betula Forte and betulin were tested across a broad range of concentrations. Betula Forte demonstrated no cytotoxicity at any of the tested concentrations, whereas betulin decreased the viability of differentiated SH-SY5Y cells at concentrations exceeding 20 µM. These findings indicate that the betulin–cyclodextrin complex effectively reduces betulin’s toxic potential at higher concentrations. Our results are in line with the study by Gonzales et al., who, while testing novel pentacyclic triterpenes, demonstrated that most components displayed no cytotoxic activity against differentiated SH-SY5Y cells within the concentration range of 0.1–10 µM [5]. It is important to note that the concentrations used for the in vitro models in this study and the dose used during the in vivo experiments in our previous report [9] were determined independently due to fundamental differences between the two experimental systems. In vitro studies were conducted under a highly controlled cellular environment, where the compound was applied directly to cultured cells. The selected concentration range (1–30 µM) for betulin and Betula Forte was based on prior literature data and preliminary cytotoxicity profiling to span subtoxic to moderately toxic levels, allowing us to observe dose-dependent cellular responses. In contrast, in vivo dosing must account for complex physiological variables. The selected in vivo dose (100 mg/kg/day) was chosen based on previous reports of similar applications of betulin in animal models. Therefore, while both dosing strategies were optimized for their respective models, a direct quantitative correlation between in vitro concentrations and in vivo dosing is not feasible without detailed pharmacokinetic and tissue distribution studies. Future studies integrating pharmacokinetic profiling and tissue-level drug quantification will be essential to bridge this gap and provide a more translational perspective. In this context, the role of cyclodextrin complexation is particularly important. According to many reports, cyclodextrins (CDs) play a crucial role in overcoming the physicochemical limitations of betulinic acid, particularly by improving its bioavailability and water solubility. Complexation with CDs has shown beneficial effects, enhancing the in vitro antiproliferative activity of betulinic acid and inhibiting tumor development in vivo [18]. In general, CD complexes can reduce the required drug dose while maintaining therapeutic efficacy [19]. Cyclodextrins are widely used in pharmaceutical formulations, both as solubility enhancers and as carriers facilitating the delivery of lipophilic drugs across the blood–brain barrier (BBB). For instance, the complexation of crocetin with γ-cyclodextrin has been shown to increase its bioavailability and BBB permeability [20]. However, BBB penetration by CDs varies depending on their type and molecular size, and many CDs exhibit limited ability to cross the BBB. This variability underscores the need for further in vivo studies to confirm the brain-targeting efficiency of the betulinic acid/betulin–cyclodextrin complex.

For the study of the neuroprotective potential of betulin and Betula Forte, low (1 µM), intermediate (5 µM), and high (10 µM) concentrations were selected. To assess whether Betula Forte and betulin could be used prophylactically as agents protecting against neurodegeneration induced by oxidative stress, we employed a pre-incubation protocol, where both components were administered 24 h before exposure to toxic concentrations of H_2_O_2_. Betula Forte, more effectively than betulin, and in a statistically significant manner, enhanced the survival of differentiated SH-SY5Y cells at all tested concentrations, even in the presence of a strong H_2_O_2_-induced effect that reduced cell viability to 20% (IC80 concentration) (Figure 2 and Table 1). Similarly, during simultaneous administration, Betula Forte provided greater protection for differentiated SH-SY5Y cells against strong oxidative stress (for H_2_O_2_ at the IC80 concentration, Figure 3 and Table 2). However, when H_2_O_2_ was administered at the IC50 concentration, Betula Forte exhibited a weaker neuroprotective effect compared to betulin (Figure 3 and Table 2). Interestingly, the effects of the tested substances do not appear to be strictly dose-dependent. In Figure 2A and Figure 3A, betulin at the concentration of 1 µM shows a stronger effect on cell viability than betulin used at the higher concentration, i.e., 10 µM. This observation may be attributed to the cytotoxic properties of betulin at higher doses, as demonstrated in Figure 1A. In contrast, Betula Forte exhibits relatively consistent potency across tested concentrations and does not show significant cytotoxicity (Figure 1B). These results suggest that the cytoprotective potential of betulin may be limited to lower concentrations by its narrower therapeutic window, while Betula Forte, possibly due to its complex composition, offers a broader safety margin allowing for more stable and predictable biological activity across different concentrations.

The results of this analysis demonstrated the neuroprotective properties of Betula Forte and, to a slightly lesser extent, betulin, primarily due to their antioxidant activity. To confirm the hypothesis, we conducted a subsequent experiment to measure the effects of both components on the levels of ROS generated by H_2_O_2_. Simultaneous administration of H_2_O_2_ and Betula Forte or betulin at low (1 µM) and intermediate (5 µM) concentrations completely prevented ROS formation, (Figure 4) suggesting that ROS inhibition is likely a key mechanism in our experimental model. The significant antioxidant potential of betulin was demonstrated in a study by Yang et al. [21], where betulin enhanced cell viability against H_2_O_2_-induced toxicity in neuron-like PC12 cells, confirming its antioxidant properties and role in neuroprotection. Supporting these results, in a study in a mouse model of asthma, betulin not only reduced ROS generation but also elevated antioxidant enzyme levels and decreased oxidative markers [22]. Similarly, in a pentylenetetrazole-induced seizure model in mice, betulin exhibited antioxidant and anticonvulsant potential by suppressing iNOS/nNOS gene expression, leading to reduced NO production [23]. In contrast, in the study by Chen et al., betulin significantly enhanced ROS production and apoptosis induction in the presence of a toxic agent, arsenic trioxide (As_2_O_3_), in the undifferentiated SK-N-SH neuroblastoma cell line [24]. The study highlighted that betulin, when combined with a potent oxidative agent such as As_2_O_3_, may shift from acting as an antioxidant to a pro-oxidant, facilitating oxidative-stress-induced apoptosis rather than mitigating it. In another study, the strong anticancer potential of betulin was demonstrated [25]. At concentrations of 2–8 μM, betulin significantly reduced cell viability and colony formation in colorectal cancer (CRC) cell lines, including CT26, HCT116, and SW620. Its anticancer activity is mediated through multiple mechanisms, including cell cycle arrest, autophagy induction, and MAPK-mediated apoptosis. Different outcomes from the cited studies and our study suggest that betulin may act as a proapoptotic agent in cancer cells, while exhibiting protective, antiapoptotic effects in stressed, differentiated, neuron-like cells. This dual behavior underscores its potential for selective therapeutic applications depending on the cellular context.

Our study aimed also to evaluate whether the neuroprotective effects of the tested substances involve the inhibition of early-stage apoptosis in differentiated SH-SY5Y neuroblastoma cells. The addition of betulin or Betula Forte to H_2_O_2_-exposed differentiated SH-SY5Y cells significantly reduced the percentage of apoptotic cells, indicating the antiapoptotic effects of both substances as part of their neuroprotective mechanism (Figure 5). Interestingly, although Betula Forte and betulin completely suppressed ROS formation in differentiated SH-SY5Y cells, cell viability declined after 24 h. Similarly, early apoptosis induced by H_2_O_2_ was not fully inhibited by either Betula Forte or betulin at tested concentrations. These findings suggest that the neuroprotective effects of both components are more complex and likely involve additional mechanisms. While preventing oxidative stress is undoubtedly crucial in our experimental model, it is likely not the sole contributing factor. Literature data, along with our previous study on a rat model of Alzheimer’s disease [9], indicate that betulin may exhibit multifaceted actions. On one hand, it may target mechanisms specific to certain conditions, such as preventing beta-amyloid formation in Alzheimer’s disease [9] or alpha-synuclein aggregation in Parkinson’s disease [26]. On the other hand, betulin might also mitigate processes common to various neurodegenerative disorders such as the downregulation of genes involved in apoptosis [26], or reduce the levels of inflammatory cytokines - including IL-1β, IL-6, and TNF-α - in the serum and hippocampus of streptozotocin-induced diabetic rats exhibiting cognitive decline [24]. In a model of cognitive decline in diabetic rats, betulin demonstrated diverse effects by improving glucose intolerance and basal learning performance. Its neuroprotective properties were associated not only with a reduction in proinflammatory cytokine levels in the hippocampus but also with restored superoxide dismutase (SOD) activity and decreased malondialdehyde (MDA) content [27].

Regarding the signaling pathways linked to the neuroprotective actions of betulin, modulation of the HO-1/Nrf-2/NF-κB pathway has been identified in diabetic rats with a cognitive decline [24], along with the PI3K/AKT pathway in AD-like pathology [28]. Recently, the impact of betulin on the DJ-1 protein has been investigated. DJ-1 is the product of a novel oncogene initially identified in Parkinson’s disease and is associated with autosomal-recessive hereditary Parkinson’s disease, also known as *Park7* or *DJ-1* [29]. The expression of the DJ-1 protein is ubiquitous in mammalian cells and is moderately expressed in central nervous system cells. It is redox-sensitive and can be significantly induced under oxidative stress [30]. Furthermore, DJ-1 serves as a neuroprotective protein with diverse functions, among which its ability to mitigate mitochondrial oxidative stress is considered paramount [31]. DJ-1 stimulates the expression of antioxidative and antiapoptotic genes, thereby activating the pro-survival Akt signaling pathway [32]. The study by Lu et al. demonstrated that betulin treatment during the acute phase of subarachnoid hemorrhage (SAH) can increase intracellular DJ-1 protein levels. This, in turn, upregulates the Akt pathway, inhibiting apoptosis and oxidative damage, thereby reducing brain edema and improving neurological outcomes [33]. These findings suggest that DJ-1 may serve as a key molecular target for betulin, linking its antiapoptotic and antioxidant effects in alleviating neurological deficits and possibly neurodegenerative disorders.

## 4. Materials and Methods

### 4.1. Reagents

Dulbecco’s Modified Eagle Medium High Glucose (DMEM-HG) was obtained from Cell Line Service (CLS, Eppelheim, Germany). Foetal bovine serum (FBS), phosphate-buffered saline (PBS), and Penicillin–Streptomycin Solution were purchased from Biowest (Nuaillé, France). Retinoic acid, hydrogen peroxide (H_2_O_2_), dimethyl sulfoxide (DMSO), Hoechst 33258 solution, and betulin were sourced from Merck (Darmstadt, Germany). The ROS Fluorimetric Assay Kit was acquired from Invitrogen (Carlsbad, CA, USA), and the Annexin V-FITC Fluorescence Microscopy Kit from BD Pharmingen (San Diego, CA, USA) via Fisher Scientific (Waltham, MA, USA). The Cell Proliferation Kit (XTT based) was from Biological Industries (Cromwell, CT, USA).

The Betula Forte preparation, consisting of a complex of betulin with cyclodextrin, is described and protected under patent No. PL241271 B1 and No. EP 3774331 A1.

### 4.2. Differentiation of SH-SY5Y Cell Line

The experiments were conducted on neuronal differentiated human neuroblastoma SH-SY5Y cells (Cell Lines Service (Eppelheim, Germany), cat. no. 300154, batch no 300154-121122). SH-SY5Y cells were grown in DMEM-HG culture medium supplemented with 10% heat-inactivated FBS and 1% Penicillin—Streptomycin Solution (PS) and maintained at 37 °C in a saturated humidity atmosphere containing 5% CO_2_. For differentiation, cells were seeded in 96-well plates at a density of 15,000 cells per well and subjected to a 7-day differentiation process using retinoic acid (RA) added to the cell culture medium at a final concentration of 10 μM according to the protocol of Jantas et al. [34]. Differentiation was carried out under conditions of 5% CO_2_ in a saturated humid atmosphere at 37 °C. One day before the experiments, the culture medium was replaced with DMEM-HG containing 1% of FBS and 1% PS. Stock solutions of the selected active substances and H_2_O_2_ were prepared in DMEM medium supplemented with 1% FBS and 1% PS.

### 4.3. Cell Treatment

For cytotoxicity measurements, differentiated SH-SY5Y cells were exposed to tested substances at concentrations ranging from 1 to 30 μM (samples were dissolved in DMSO to create stock solutions of 2.5 mg/mL). To evaluate the protective effects of the tested substances toward differentiated SH-SY5Y cells, two experimental approaches were adopted, i.e., pre-incubation and co-incubation. The aim of the pre-incubation and co-incubation variants was to determine whether the active substances (betulin and Betula Forte) provide protective effects on differentiated neuroblastoma cells either before exposure to a neurotoxic agent or during simultaneous exposure to a toxic agent (i.e., H_2_O_2_) and active substance, respectively. In both experimental variants, H_2_O_2_ solutions were prepared at concentrations that reduced the viability of differentiated neuroblastoma cells to 50% (IC50) or 20% (IC80).

### 4.4. Measurements of Cell Viability

To determine the IC50 and IC80 values for H_2_O_2_, the cytotoxicity of the H_2_O_2_ solution in a specified range toward differentiated neuroblastoma cells was measured with the use of an XTT assay. A broad range of concentrations of active substances was also examined during pilot experiments to identify non- and sub-cytotoxic concentrations (cell viability above 70%). The determination of cellular metabolic activity with the XTT compound is based on the reduction of the yellow XTT salt (sodium salt of 2,3-bis(2-methoxy-4-nitro-5-sulfophenyl)-2H-tetrazole-5-carboxanilide) to formazan, resulting in an orange solution. This reduction occurs only in living cells with intact metabolism and respiratory chain. The conversion of XTT salt by the enzyme mitochondrial dehydrogenase is facilitated by the intermediate electron acceptor PMS (1-methoxy-5-methylphenazine methyl sulphate). The intensity of the resulting color is linearly proportional to the number of viable cells and is measured spectrophotometrically at 450 nm, with a reference reading at 630 nm.

During pilot experiments, differentiated cells were exposed to betulin and Betula Forte for 24 h under standard culture conditions (5% CO_2_, 37 °C, and 95% humidity). After the incubation period, activated XTT reagent was added to the medium, and the resulting absorbance was measured after 4 h at 450 nm and 630 nm using a microplate reader, Victor X4 (Perkin Elmer, Waltham, MA, USA).

**Figure 6:**
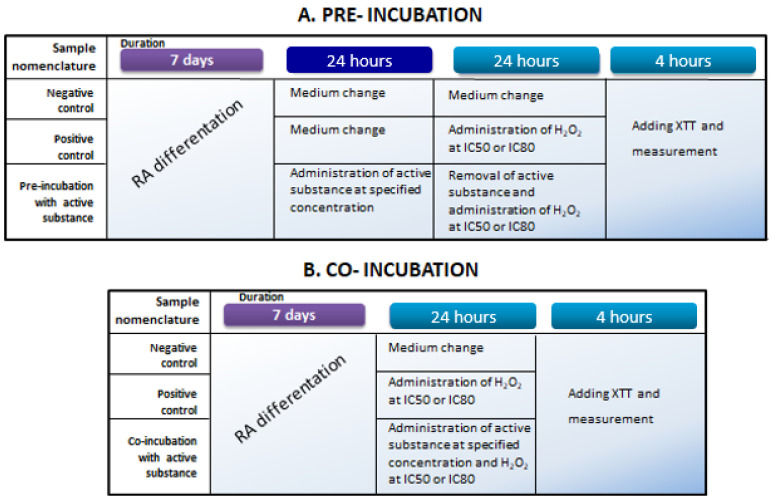
Schematic representation of pre-incubation and co-incubation experimental design for the SH-SY5Y cell line, along with sample nomenclature.

In the pre-incubation variant (Figure 6A), after differentiation, SH-SY5Y cells were exposed to active substances for 24 h at concentrations of 1, 5, and 10 µM. The next day, the medium was removed from each well to eliminate the tested samples, and 100 μL of fresh cell culture medium containing H_2_O_2_ at a final concentration corresponding to IC50 or IC80 was added for another 24 h of incubation. Cells not subjected to any stimulation served as a negative control, while those exposed to H_2_O_2_ alone at the selected concentrations served as a positive control. The following day, an XTT assay was performed according to the previously described procedure.

In the co-incubation variant (Figure 6B), after differentiation, SH-SY5Y cells were exposed to active substances at concentrations of 1, 5, and 10 µM along with H_2_O_2_ at concentrations corresponding to IC50 and IC80, simultaneously for 24 h in standard culture conditions. Cells not subjected to any stimulation served as a negative control, while those exposed to H_2_O_2_ alone at the selected concentrations served as a positive control. After the incubation period, an XTT measurement was performed as described above.

### 4.5. Labeling of Differentiated SH-SY5Y Cells with Annexin V—FITC

The early apoptosis assay is based on the interaction of Annexin V with phosphatidylserine (PS), an amino phospholipid that is present on the inner side of the cell membrane in healthy cells. In the early stages of apoptosis, PS is exposed to the external environment (on the outside of the cell membrane) and can serve as a marker to detect apoptosis when bound to FITC-labeled Annexin V.

Early-phase apoptosis cells were labeled using an Annexin V-FITC Fluorescence Microscopy Kit (BD Pharmingen). For quantitative analysis of the early phase of apoptosis, cell nuclei were also stained with a Hoechst marker.

SH-SY5Y neuroblastoma cells for Annexin V-FTC staining were seeded, differentiated, and treated with Betula Forte/betulin in a configuration defined as co-incubation for 24 h. The samples were designated as KN (negative control), H_2_O_2_ IC50 (cells exposed to H_2_O_2_ at a concentration reducing their viability to 50%), Bet 1 µM and 5 µM (cells incubated with betulin at concentrations of 1 and 5 µM), and Bet 1 µM and 5 µM + H_2_O_2_ IC50 (simultaneous incubation of cells with betulin at selected concentrations and with H_2_O_2_ at the IC50 concentration). Analogous measurements were performed for Betula Forte.

Briefly, after 24 h of cells’ co-incubation with active substances and H_2_O_2_, cells were washed with PBS Buffer and subsequently with Annexin V Binding Buffer. Next, cells were stained with Annexin V-FITC for 15 min at room temperature according to the manufacturer’s protocol. For the next 5 min, a Hoechst marker was added at a final concentration of 5 µg/mL at RT in the dark. After staining, differentiated cells were washed with Annexin V Binding Buffer and imaged on an InCell Analyzer 2000 automated fluorescence microscope (GE Healthcare Life Sciences, Pittsburgh, PA, USA). Analysis was performed using InCell Developer software 1.92 (GE Healthcare Life Sciences, Pittsburgh, PA, USA). Apoptotic cells were identified based on the fluorescence intensity of the FITC channel, and the total number of cells, both viable and apoptotic, was estimated by staining cell nuclei with Hoechst. Results were expressed as the percentage of apoptotic cells.

### 4.6. ROS Detection

The assessment of oxidative stress in differentiated SH-SY5Y cells was conducted using an ROS Fluorimetric Assay Kit (Invitrogen, EEA019), which contains a fluorescein derivative, specifically 2′,7′-dichlorofluorescein diacetate (DCFH-DA). The principle of the test is based on a two-step reaction that converts a primary non-fluorescent compound into a fluorescent form. In the first step, DCFH-DA undergoes intracellular deacetylation by esterases present in living cells, followed by oxidation of the reduced fluorescein compound by reactive oxygen species (ROS). The oxidized DCF emits green fluorescence, and the intensity of this fluorescence is proportional to the amount of ROS present in the cell.

The experiment was conducted in 2 h of the co-incubation variant for H_2_O_2_ concentrations corresponding to IC50 values, and for betulin and Betula Forte concentrations of 1 μM and 5 μM. As a positive control, H_2_O_2_ was used at a concentration resulting in a 50% reduction in cell viability (IC50), and tert-butyl hydroperoxide (TBHP) was included from the assay kit and used at a concentration of 250 μM.

Briefly, after 2 h of cells co-incubation with active substances and H_2_O_2_, DCFH-DA was added to the culture at a concentration of 20 μM, and cells were incubated for 1 h at 37 °C and 5% CO_2_. After this period, the culture was gently pipetted to detach the cells from the cell culture wells, collected into Eppendorf tubes, and centrifuged for 2 min at 130× *g*. After centrifugation, the supernatant was carefully removed, and the cell pellet was resuspended in 100 μL of HBSS buffer with Ca^2+^ ions. A fluorescence measurement was performed on a 96-well plate using a Victor X4 microplate reader (Perkin Elmer, Waltham, MA, USA) in fluorescence mode with an excitation wavelength of 485 nm and an emission wavelength of 535 nm. Results were presented as the percentage of cells with ROS activation relative to the negative control ROS level.

### 4.7. Data Analysis

Results are presented as mean values ± standard deviation (SD) for at least three independent biological replicates (the number of biological replicates for each experiment was indicated below graphs). Statistical significance was assessed with GraphPad Prism 6 software using a one-way analysis of variance (ANOVA) and Tukey’s post hoc test for multiple comparisons to evaluate statistical significance between groups. Values were considered statistically significant at * *p* < 0.05, ** *p* < 0.01, *** *p* < 0.005, and **** *p* < 0.001. Similarly, statistical significance was indicated versus the positive control (#).

## 5. Conclusions

Both betulin and its cyclodextrin complex, Betula Forte, enhance the survival of neuron-like SH-SY5Y cells exposed to oxidative stress in pre-incubation and co-incubation models with Betula Forte showing superior efficacy under severe oxidative stress.

This neuroprotective effect is primarily mediated through the antioxidant and antiapoptotic activities of betulin and Betula Forte.

Future research should further explore the molecular targets and signaling pathways involved in the neuroprotective effects of betulin and its improved formulations, as well as their therapeutic efficacy in diverse neurodegenerative disease models.

## Figures and Tables

**Table 1:** Relative changes in cell viability versus the positive control (H_2_O_2_) calculated from the cytotoxicity results for the pre-incubation variant.

Pre-Incubation			
H_2_O_2_ concentration: IC50	IC_50_ + 1 μM	IC_50_ + 5 μM	IC_50_ + 10 μM
Betulin [μM]	15.79	11.08	9.24
Betula Forte [μM]	17.54	16.49	20.52
**H_2_O_2_ concentration: IC80**	**IC_80_ + 1 μM**	**IC_80_ + 5 μM**	**IC_80_ + 10 μM**
Betulin [μM]	15.92	8.41	3.93
Betula Forte [μM]	15.15	15.07	8.25

**Table 2:** Relative changes in cell viability versus the positive control (H_2_O_2_) calculated from the cytotoxicity results for the co-incubation variant.

Co-Incubation			
H_2_O_2_ concentration: IC50	IC_50_ + 1 μM	IC_50_ + 5 μM	IC_50_ + 10 μM
Betulin [μM]	27.6	18.2	1.8
Betula Forte [μM]	3.3	10.7	10.9
**H_2_O_2_ concentration: IC80**	**IC_80_ + 1 μM**	**IC_80_ + 5 μM**	**IC_80_ + 10 μM**
Betulin [μM]	−1.8	7.7	3.1
Betula Forte [μM]	13.6	11.9	17.4

## Data Availability

The original contributions presented in the study are included in the article; further inquiries can be directed to the first author and corresponding authors.
